# Views of People With High and Low Levels of Health Literacy About a Digital Intervention to Promote Physical Activity for Diabetes: A Qualitative Study in Five Countries

**DOI:** 10.2196/jmir.4999

**Published:** 2015-10-12

**Authors:** Alison Rowsell, Ingrid Muller, Elizabeth Murray, Paul Little, Christopher D Byrne, Kristin Ganahl, Gabriele Müller, Sarah Gibney, Courtney R Lyles, Antonia Lucas, Don Nutbeam, Lucy Yardley

**Affiliations:** ^1^ Department of Psychology Faculty of Social and Human Sciences University of Southampton Southampton United Kingdom; ^2^ Faculty of Health Sciences University of Southampton Southampton United Kingdom; ^3^ Primary Care and Population Sciences Faculty of Medicine University of Southampton Southampton United Kingdom; ^4^ Research Department of Primary Care and Population Health University College London London United Kingdom; ^5^ Nutrition and Metabolism Faculty of Medicine University of Southampton Southampton United Kingdom; ^6^ Southampton National Institute for Health Research Biomedical Research Centre University Hospital Southampton Southampton United Kingdom; ^7^ Ludwig Boltzmann Institute of Health Promotion Research Vienna Austria; ^8^ Centre for Evidence-based Healthcare University Hospital Carl Gustav Carus Technical University Dresden Dresden Germany; ^9^ UCD School of Business University College Dublin Dublin Ireland; ^10^ Division of General Internal Medicine at San Francisco General Hospital University of California San Francisco, CA United States; ^11^ Office of the Vice-Chancellor University of Southampton Southampton United Kingdom

**Keywords:** health literacy, digital intervention, diabetes, qualitative, physical activity

## Abstract

**Background:**

Low health literacy is associated with poor health-related knowledge, illness self-management, health service use, health, and survival, and thus addressing issues related to low health literacy has been highlighted as a pressing international priority.

**Objective:**

To explore views of a digital health promotion intervention designed to be accessible to people with lower levels of health literacy, in particular examining reactions to the interactive and audiovisual elements of the intervention.

**Methods:**

Qualitative think-aloud interviews were carried out with 65 adults with type 2 diabetes in the UK, Ireland, USA, Germany, and Austria, with purposive sampling to ensure representation of people with lower levels of health literacy. Inductive thematic analysis was used to identify common themes. We then systematically compared views in subgroups based on country, health literacy level, age, gender, and time since diagnosis.

**Results:**

Most participants from the chosen countries expressed positive views of most elements and features of the intervention. Some interactive and audiovisual elements required modification to increase their usability and perceived credibility and relevance. There were some differences in views based on age and gender, but very few differences relating to health literacy level or time since diagnosis.

**Conclusions:**

In general, participants found the intervention content and format accessible, appropriate, engaging, and motivating. Digital interventions can and should be designed to be accessible and engaging for people with a wide range of health literacy levels.

## Introduction

Addressing the problem of low health literacy has been highlighted as a pressing international priority, as it is associated with poor health-related knowledge, illness self-management, health service use, health, and survival [1]. The concept of health literacy has evolved to refer to “the knowledge, motivation and competences to access, understand and apply health information” [2]. Health literacy levels tend to be lower in those with less education, lower incomes, and minority ethnics groups [1]. Health literacy is a product of the interaction between the individual and his or her environment, which includes the health care resources available to him or her; designing health care materials to be accessible and easily comprehensible can reduce the literacy burden, and improve health literacy by helping people to understand and implement health-related advice [3].

Many people have difficulty accessing face-to-face diabetes self-management education, due to barriers such as work, caring responsibilities, disability, cost, and lack of transport [4], and these problems are more common among those with less education [5]. The rapid growth in delivery of health promotion and health care by means of digital interventions offers one possible solution to this challenge; digital interventions can be accessed conveniently at home and have the potential for wide reach at low cost, and so could reduce health disparities [6]. However, there is a risk that digital interventions could increase health inequalities due to a “digital divide” in both access to the Internet and confidence and skills to use the Internet for self-management of health [7]. Internet access is now increasing rapidly among all social groups, but low literacy levels may continue to pose barriers to understanding and applying online information. For example, one study of diabetes websites found that 86.9% of materials would be too difficult to read for an average adult [8], and from early studies of Internet-delivered support for people with diabetes there is evidence of lower usage by those with lower income and education [9,10].

Interventions to reduce the literacy burden and improve health literacy have included using simple language; information presented in audio, audiovisual, or pictorial formats; (when Internet delivered) employing tailoring of content to individual’s needs; and other forms of interactivity. Reviews of the effectiveness of such interventions for general public and mixed patient populations [11-15] and for diabetes [16-18] suggest that these approaches show promise for some outcomes, but that overall the evidence is weak and inconclusive. Few studies have been theory based, and it remains unclear exactly which elements of such interventions improve which outcomes. The authors of these studies call for more research to identify the effective components and mechanisms of interventions to improve health literacy and to examine whether these differ for people with different levels of health literacy [11,14,15]. Empirical evidence that addresses the latter question is crucial to inform those delivering health care about whether it is necessary to create different versions of online interventions to engage different sectors of the population; such evidence will consequently have wide-ranging practical and resource implications.

As part of the Diabetes Literacy project [18], our aim was to address this evidence gap by examining how people with varying levels of health literacy viewed features of a digital intervention we developed to explore how best to increase accessibility and acceptability of health promotion resources for people with lower levels of health literacy. Our primary aim was therefore to examine whether our website design was acceptable and engaging for people with differing levels of health literacy. Because digital delivery permits interactive and audiovisual presentation of advice, that could potentially help to overcome difficulties due to low literacy, we were also particularly interested in exploring participants’ views of these features of the intervention.

##  Methods

###  Intervention

**Figure 1:**
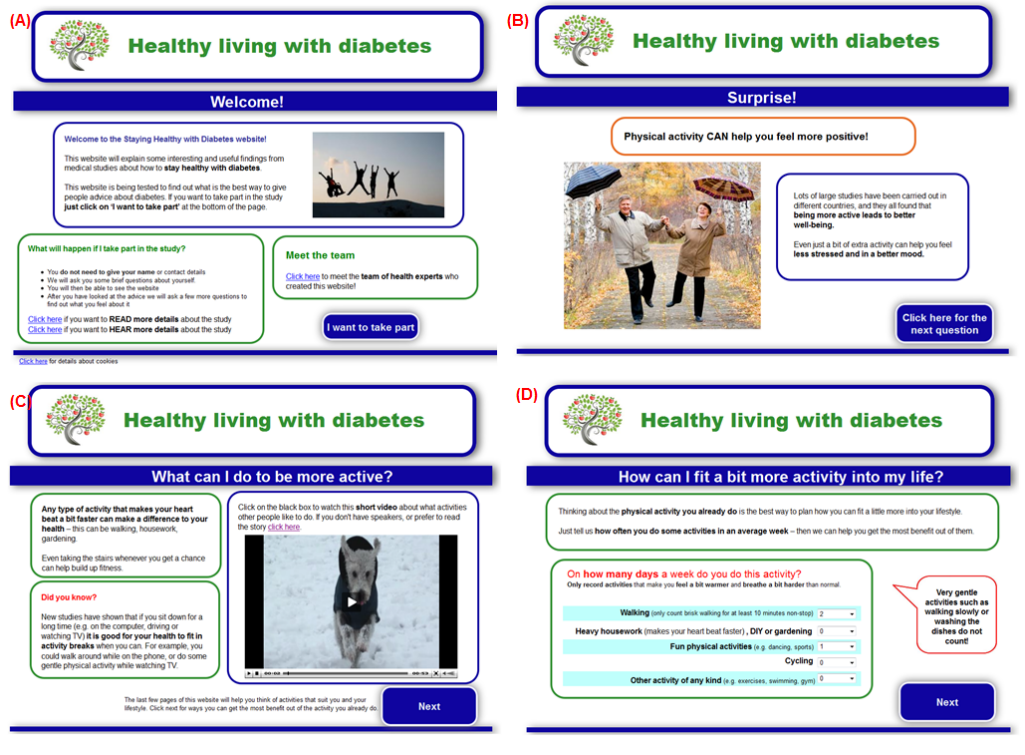
Screenshots illustrating elements of the Healthy Living with Diabetes website. (A) Example of positive visual image on welcome page. (B) Example of quiz feedback with positive visual image. (C) Example of audiovisual sequence. (D) Example of interactive physical activity planner.

The intervention was a website designed to motivate people with diabetes to increase their levels of physical activity. The interactive features of the website comprised tailoring of images and advice based on user responses to questions (eg, about the user’s age, concerns about physical activity, current activity levels), a quiz, and a physical activity planner. The audiovisual features included positive images and audiovisual sequences illustrating lifestyle physical activities. We followed established good practice for designing written medical information and making it accessible to people of all literacy levels [11,19], and used design principles that have been shown to increase accessibility of websites for people with cognitive impairment and limited computer literacy [20]. See Textbox 1 for further details of the elements of the intervention, and Figure 1 for example screenshots.

The intervention was developed in consultation with an expert panel of patient representatives, clinicians, and behavioral scientists. We employed the person-based approach to intervention development [21], which grounds intervention design in a rigorous, in-depth understanding of the psychosocial context of the target user population. A key element of the person-based approach to intervention development is to use iterative, inductive qualitative research to explore users’ views of the intervention and then modify the intervention to optimize acceptability and engagement. Consequently, small changes to the website were made throughout data collection. These included, for example, changing the format of the physical activity planner to improve usability, changing wording to improve comprehension, and substituting images that were disliked. The intervention was developed using *LifeGuide* software, a platform for developing online behavior change interventions that allows researchers to easily translate and modify the intervention [22]. The website was initially developed in English for evaluation in the UK, Ireland, and USA, and then translated for evaluation in Germany and Austria (all text of the translated versions was checked for accuracy by the researchers in these countries).

**Elements and design features of the Healthy Living with Diabetes intervention.**

Interactive elements of the intervention

- Delivery of information about the health benefits of physical activity for people with diabetes (and health risks of inactivity) in the form of an interactive “fun” quiz.
- Tailored advice in response to questions about current physical activity level and concerns about physical activity (eg, barriers such as cost, health problems).
- Tailoring of images based on users’ reported age.
- A physical activity planner that enabled users to create personal plans for increasing physical activity, building on their current activities.

Audiovisual elements of the intervention

- Positive visual images throughout the intervention.
- Audiovisual sequences modeling people undertaking a range of physical activities: these comprised a narrative illustrated by a sequence of photographs with a voice-over, and had an informal tone intended to suggest real-life scenarios.
- Option to access details of study aims and procedures in audio format.

Good practice design features of the intervention

- To maximize accessibility to those with low levels of literacy, text as short as possible, suitable for reading age of 12.
- To maximize accessibility to those with low levels of computer literacy, simple page layout and navigation.
- Advice appropriate for those with low current levels of physical activity, limited time, motivation and resources, and health problems (ie, promoted gradual increase in preferred lifestyle-compatible activities).

### Design, Participants, and Procedure

Qualitative interviews were carried out with adults with type 2 diabetes in the UK, Ireland, USA, Germany, and Austria. Full details of all study procedures, participants, and analyses are published online [23]. The UK sample was used as the core group for intervention development and evaluation, and data collection in the other countries was used to explore whether views differed in different countries and settings. Participants were recruited from primary care, community settings, and diabetes support groups, using advertisements, letters, and personal invitations; for full details see [23]. We purposively sampled to include people with lower levels of health literacy by recruiting from areas and clinics with high levels of socially deprived patients. We also used diversity sampling to ensure that we had a broad balance of both genders, and different ages and time since diagnosis.

Qualitative think-aloud methods [24] were used to gain a thorough, in-depth understanding of user experiences, perceptions, and views of the intervention, followed by a semistructured interview, allowing specific domains of user experience to be explored. Experienced researchers who received standardized training on conducting think-aloud interviews interviewed participants in their own language in each country, in a variety of settings in clinics and the community. Participants also completed a brief questionnaire to measure age, gender, time since diagnosis, and health literacy. Health literacy was measured using the validated single item “how often do you have problems learning about your condition because of difficulty understanding written information?”[25], allowing participants to be categorized as having high, intermediate, or low levels of health literacy. To enable us to validate this single-item, categorical measure of health literacy, we examined its correlation with a more comprehensive measure of all dimensions of health literacy, the 16-item version of the European Health Literacy Survey Questionnaire (HLS-EU-16) [26]; this was completed by all participants after the interviews.

###  Analysis

Audio recordings of interviews were transcribed verbatim, translated into English where necessary, and checked for accuracy by the researcher who carried out the interview. Few substantial differences in views between countries were identified (see [23] for details), allowing the data to be pooled to give a larger sample for the remaining comparisons. Inductive thematic analysis was used to identify recurring themes through close examination of the data [27,28]. We also used analytic techniques from grounded theory to increase the rigor of our analysis, including line-by-line coding and constant comparison [29]. First, interview transcripts were read and reread to ensure a high level of familiarity with the data before line-by-line coding of the initial 3 interviews. A provisional coding manual (see [23] for details) was then created to define emerging codes and themes before these codes were applied to the remaining transcripts. The coding manual was developed iteratively and revised throughout the coding process to ensure the codes adequately reflected the data. The coding manual was discussed and agreed upon by core members of the research team (AR, IM, and LY) at various stages of the coding process, and inter-rater agreement between AR and IM was obtained for all the final coded data.

The relatively large sample size (for qualitative research) allowed us to carry out a second stage of analysis to explore whether any differences could be identified between specific subgroups of participants. We did this by creating tables to systematically compare the occurrence and content of themes across subgroups (see ). First, country comparisons were carried out to examine whether there were differences in the data between countries that might preclude pooling the data across countries for analysis. Only the data from the German sample appeared systematically different, largely in terms of less views being expressed on most topics. This finding may have been related to deviations from the interview protocol evident in the transcripts (eg, a less open-ended interviewing style than in other countries, omission of substantial parts of the interview schedule). In view of this difference, the 6 German interviews were excluded from the subsequent subgroup analyses. Further subgroup analyses on the remaining pooled data comprised comparisons based on level of health literacy (low, intermediate, or high), age (under or over 60 years), gender, and time since diagnosis (less or more than 5 years).The cutoff points were chosen so as to create roughly equal groups (to maximize the sample size in each) that were likely to differ in terms of experiences of diabetes (based on the research team’s clinical experience of diabetes patients). Because of the small size of the subgroups and large number of comparisons, to avoid overinterpretation of minor differences we adopted a criterion of only reporting comparisons where the differences observed between the groups compared were based on at least five people (between country comparisons) or 30% (n=19) of participants (pooled data), or suggested a consistent trend from high to low health literacy (even if not quite reaching our criterion within the specific subgroups).

##  Results

### Participant Characteristics

In total, 65 participants were interviewed for this study, comprising 35 from the UK, 8 from USA, Ireland and Austria, and 6 from Germany (Table 1). There were 37 (57%) men and 28 (43%) women, with a mean age of 62 years (range 37–79), and 40 (62%) participants had been diagnosed with type 2 diabetes for 5 years or more. As classified by our single-item measure, 38 (58%) participants had high health literacy, 18 (28%) had intermediate health literacy, and 8 (12%) had low health literacy (1 was “unknown”). The single-item measure was highly correlated with the HLS-EU-16 in a subsample sample (*r*=.65, n=35, *P*<.001), providing reassurance that our single-item categorization assessing literacy problems in a health context was associated with health literacy problems in a range of other domains. Participant characteristics were similar across all countries, with the exception of Germany (all German participants had high levels of health literacy and all but 1 person had diabetes for over 5 years).

**Table 1:** Participant characteristics.

Participant characteristics		Country					
		UK	USA	Ireland	Austria	Germany	Overall
**Age (in years)**							
	Mean	58	57	66	60	67	62
	Range	44-75	49-64	37-77	42-79	48-77	37-79
	≥60 years	18	3	6	3	4	34
	<60 years	17	5	2	5	2	31
**Time since diagnosis**							
	<5 years	14	4	4	2	1	25
	≥5 years	21	4	4	6	5	40
**Gender**							
	Male	18	3	6	6	4	37
	Female	17	5	2	2	2	28
**Health literacy level**							
	High	16	4	6	7	5	38
	Intermediate	13	2	2	0	1	18
	Low	6	1	0	1	0	8
	Not known	0	1	0	0	0	1
**Ethnicity**							
	White/Caucasian	32	2	7	8	6	55
	Black/African/Caribbean	2	2	0	0	0	4
	Asian	1	0	1	0	0	2
	Other (mixed)	0	4	0	0	0	4
Total sample		35	8	8	8	6	65

###  Themes Emerging From Pooled Analysis

Thematic analysis of the data pooled across countries generated 40 codes, which we organized into 3 main themes, namely, (1) general reactions to website content and format; (2) reactions to interactive features; and (3) reactions to audiovisual features.

### General Reactions to Website Content and Format

The majority of users described the level of information in the intervention as appropriate and easy to understand, without being patronizing (Table 2). The website was also described as accessible and as more user friendly than other resources they had encountered.

I like the whole thing. It didn’t blind you with science; it didn’t treat you as a total idiot.

UK, male, under 60 years old, under 5 years’ diagnosis, high HL

It’s written in a way that’s not—because I mean I’m not good at reading and stuff like that—but it’s written in a way that I can understand. You know, sometimes you look at, whether it’s books or websites or whatever, and sometimes you’re reading and you think “What are they talking about?”

UK, female, under 60 years old, over 5 years’ diagnosis, low HL

The thing is that all the knowledge I received during hours of lectures [at “wellness clinics”] is briefly put together here, so really, really well done for this!

Austria, male, under 60 years old, under 5 years’ diagnosis, low HL

No, it’s very straight forward and compact. What you need to know is there.

Austria, male, under 60 years old, over 5 years’ diagnosis, high HL

Participants frequently spoke appreciatively of acquiring new information through the website and the positive framing of health information was described as encouraging.

Yeah, I know they say physical, mental activity can, can help you stay healthy [reads to self], “twice,” woah, “twice as likely!” Get out, really? I don’t remember ever hearing that one.

USA, female, over 60 years old, over 5 years’ diagnosis, high HL

To find out that Alzheimer’s thing, yes that was quite shocking, a sit up and take notice moment.

UK, male, under 60 years old, over 5 years’ diagnosis, intermediate HL

A number of people discussed their intention to increase their physical activity as a direct result of what they had learned or seen in the website.

Interesting, I did not know that—so it's almost like, if I learn nothing else from this survey, I need to start my physical activity regime.

USA, male, over 60 years old, under 5 years’ diagnosis, intermediate HL

Specific intervention features described as motivating included the planner and audiovisual sequences as well as learning new health information through the quiz. Details of how participants viewed these features are given below.

**Table 2:** Reactions to website content and format.

Subtheme	Content
Information novelty	Almost all participants mentioned learning new, often surprising information (particularly about the benefits of physical activity for preventing Alzheimer’s disease and for healthy liver function)
Level of website advice	Most participants felt that the advice was delivered at the right level—easy to understand but not patronizing
Views of website advice	The vast majority of participants endorsed the advice given by the website.
	Many were surprised and some skeptical about the information that physical activity is more important than controlling blood sugar levels for preventing complications from diabetes.
	The humor in the website was mainly appreciated by the minority who commented on it.
Views on website appearance	Most participants commented that they found the website clear and easy to navigate.
	Some expressed a desire for greater simplicity in presentation and less text.
Effects of website on motivation	Most participants (though not all) found the website generally motivating.
	All elements of website content were described by some participants as motivating them to engage in greater physical activity.

### Reactions to the Interactive Features

Many participants commented that the interactive features of the website were engaging and motivating (Table 3), although some chose to skip sections that did not appeal to them. Most were positive about the quiz section, describing it as fun, relevant, helpful, interesting, and a preferable way of learning new information.

I’m getting really curious now to see the answers [both laugh], it’s lovely, it’s not boring, the whole Web thing is very good. It’s very interesting.

Ireland, female, over 60 years old, over 5 years’ diagnosis, high HL

I liked the quizzes, I liked that—you know—it’s nice to have something you can use, interact with and join in with.

UK, female, under 60 years old, under 5 years’ diagnosis, low HL

If I was doing this on my own I would skip this bit [quiz section], I’m bored with that now, because it's treating me like a child. I want the information but I don't like the way it's given to you.

UK, male, under 60 years old, under 5 years’ diagnosis, low HL

Participants described valuing the instant tailored feedback, and often commented on encouragement provided by the positive framing of this advice.

Woo hoo—“doing enough physical activity to keep healthy”—now says star pupil actually, that’s a bonus.

UK, female, over 60 years old, over 5 years’ diagnosis, low HL

Well done, you got it right, you got something right. See, they love me. Finally! [laughs] They love me.

USA, female, over 60 years old, under 5 years’ diagnosis, unknown HL

However, participants had more mixed responses to the activity planner, some finding it cumbersome and difficult to complete, particularly (but not exclusively) before modifications were made to simplify it. For example, the original planner required participants to specify the activity type and the amount of time spent on the activity on the same page. Many participants found this difficult to navigate, and the planner was subsequently separated onto 2 pages.

**Table 3:** Reactions to the interactive features.

Subtheme	Content
Views of the interactive quiz	Most comments on the quiz were that it was enjoyable and informative.
	Some found it irritating, and disliked the humorous “trick” questions (ie, that physical activity would not improve hearing or alcohol consumption).
Views of tailored feedback	Most comments on the feedback were that participants appreciated getting immediate, positive feedback.
	Participants noted that actively engaging with the website kept their attention.
Views on the interactive activity planner	Some participants found using the planner difficult, due mainly to uncertainty about (1) how to estimate their activity level and (2) how to enter data into the planner (before simplification of the planner)

### Reactions to the Audiovisual Features

The vast majority of participants found the images acceptable or liked them overall (Table 4).

These pictures are quite nice as well, you know, they are happy pictures—photographs of elderly people, probably my age, dancing with umbrellas.

Ireland, male, over 60 years old, over 5 years’ diagnosis, high HL

A few participants mentioned disliking certain images, although the images that people disliked varied. For example, some younger participants could not relate to images of older people; consequently, the intervention was modified to tailor all images by age.

Most participants also enjoyed the audiovisual sequences, appreciating the informal style and relating positively to the stories and example activities these sequences narrated.

The videos were really, really good...informative.

USA, male, over 60 years old, under 5 years’ diagnosis, low HL

It was good, straight to the point, because it’s like, not everybody likes going to the gym and that. It’s too expensive, too hot to try to stay and that, and the person that was speaking was really clear.

UK, male, under 60 years old, over 5 years’ diagnosis, low HL

Some participants commented positively on the use of humor in the main audiovisual sequence (which showed pictures of snowboarding and deep sea diving as examples of impractical forms of exercise before suggesting walking as more feasible) but some found it confusing or inappropriate. In addition, some people disliked particular scenarios that they found unconvincing or irrelevant:

I don’t like it at all. It doesn’t fit the pictures, there’s an old man who says he’s picking up his children. He says his day is hectic yet he can go shopping before work and during lunch. It doesn’t fit.

Germany, male, under 60 years old, over 5 years’ diagnosis, high HL

Some of the scenarios therefore required modification for different cultures or age groups; for example, a scenario that suggested playing football was changed to baseball for US participants.

**Table 4:** Reactions to the audiovisual features.

Subtheme	Content
Views of visual images in the website	Overall, use of images in website appreciated as positive, attractive.
	Images of walking and family activities generally appreciated, medical illustrations mainly well received.
	Reactions to some images mixed (prior to modification), for example, images of older people, wheelchair activity, alcohol.
Views of audiovisual sequences in the website	Most participants were very positive about the audiovisual sequences, most (though not all) liking the informal style, and finding them engaging, funny.
	Most people enjoyed relating to stories that they saw as realistic, helpful examples of lifestyle activity.
	Negative comments were often based on seeing specific content (prior to modification) as unrealistic or irrelevant to the participant’s own situation (eg, due to cultural or lifestyle differences or activity preferences).

###  Subgroup Comparisons

We found few differences in how people with high, intermediate, or low levels of health literacy viewed the website. The only differences that emerged were that people with higher health literacy were most likely to comment that they found the website content easy to understand, mention features of the website they found motivating, discuss how the interactivity maintained their attention, and express appreciation of the visual images. There were also few systematic differences in views linked to country, although there were some culture-specific preferences (as noted earlier); for example, the Austrian participants tended to dislike the audiovisual sequences, commenting negatively on the use of speakers with German accents.

With regard to other subgroup comparisons, women were more likely than men to voice their intention to be more physically active as a result of viewing the website. Women tended to express much more positive views of the quiz than men did; some men found it tedious or irrelevant. Women were also more positive about the audiovisual sequences, describing them as engaging and discussing relating to the characters and stories. Women, however, were less technically confident in completing the interactive planner. There were age differences in the activities preferred; those aged over 60 mainly intended to do more walking, whereas younger participants also spoke about cycling and swimming. Participants over 60 years of age and those who had been diagnosed for longer were more positive about the audiovisual sequences (particularly the walking stories) and the idea of exercising at home. This age group was also more likely to mention that they found the level of information in the website straightforward, helpful, or pitched at the correct level.

## Discussion

### Preliminary Findings

The main aim of this study was to investigate whether it is possible to design a digital intervention that is acceptable and engaging for people with varying levels of health literacy. Our findings are encouraging; most participants from most countries expressed positive views about most elements of our digital intervention. Very few people found the accessible format patronizing or the information provided inadequate or inappropriate. Despite concerns frequently expressed about whether it is possible to appeal to a wide and diverse target population, it is not unusual to find that materials designed to be accessible to those with lower levels of literacy and health literacy are also liked by those with higher literacy levels [30-32].

Although most participants had mainly positive reactions to the interactive and audiovisual presentation of advice, it was necessary to iteratively modify these based on participant feedback to optimize acceptability and feasibility. There were also differences in reactions to these elements of the intervention relating to age, gender, and cultural context. We were only able to evaluate views of a very limited set of digital materials, and it is quite possible that the views expressed were specific to these particular resources. For example, reactions to professionally produced videos of patients or actors might be entirely different from reactions to our amateur-style audiovisual sequences. Nevertheless, some previous research has also noted that it can be difficult to produce audiovisual narratives that precisely match the psychosocial context of all users [33]. It appears that audiovisual materials may require particularly careful development, with attention to the sociocultural context of target users, to ensure that they are perceived as convincing and relevant [21,34].

### Limitations

The method we used for comparing views across subgroups is unusual; it can be regarded as an extension of the constant comparison technique employed in grounded theory, but with more systematic and explicit assessment of the frequency with which views were expressed in different subgroups. Because these subgroups were too small to permit reliable quantitative evaluation, it is not possible to interpret the trends observed in our data as definitive evidence for the presence or absence of group differences. Nevertheless, the consistent absence of any variations in views clearly associated with health literacy and time since diagnosis, despite clear differences based on age and gender, suggests the latter may have had a more important influence on reactions to the intervention. However, we were unable to recruit many participants with the lowest levels of health literacy, and the sample size in each country was small. Consequently, this study was only able to identify substantial differences in views due to country or very low health literacy (ie, views that would be expressed by most people in these subgroups). Most participants were also white, and these findings may therefore not be generalizable to people from other ethnic backgrounds. A further limitation in our ability to fully investigate the perspective of users with lower levels of health literacy is that these participants provided fewer comments about the website and our analysis of the articulated views of users was unable to capture nonverbal indications of accessibility or engagement barriers, such as pauses and silences. Further research into the views of people with very low health literacy is required.

It is important to be aware that, despite our best efforts to encourage participants to freely express negative views, some participants may have been reluctant to do so. For example, it is possible that women did not find the intervention more engaging than men, but were less willing to express negative views of it. It is also important to remember that for the purpose of health promotion it is necessary but not sufficient for an intervention to be acceptable and engaging—it must also be effective in achieving the intended behavioral outcomes. For this reason, we are now undertaking a large trial to test the effectiveness of our digital intervention for promoting health literacy improvement (in knowledge, understanding, and self-efficacy) and behavioral intentions with regard to increased physical activity.

### Conclusion

Most participants found the intervention generally acceptable and engaging. Surprisingly, reactions were similar—and equally positive—among those with higher and lower levels of health literacy. This finding has importance for the reach and cost effectiveness of digital health care, because it suggests that it may not be necessary to develop multiple versions of interventions for people with differing levels of health literacy. However, our sample did not include people with the very lowest levels of health literacy, who may well require different online or offline interventions.

Some marked variations in preferences for how advice was presented did emerge; for example, many people particularly appreciated the interactive quizzes and audiovisual sequences, whereas a minority strongly disliked them. These variations in preferences were partly linked to age, gender, and culture but were not closely mapped onto demographic and psychosocial characteristics, making it difficult to prescribe exactly what format should be used for which population subgroup. This suggests that perhaps a major benefit of Internet delivery of health promotion is that it is possible to offer recipients a choice of formats, allowing them to self-select those that they find most accessible, attractive, and useful. This approach permits users to engage in what has been termed “self-tailoring” [35], in contrast to the pre-emptive tailoring to major preferences in target groups that intervention developers must use when constrained by the page limits of printed materials. Offering users a choice of how they engage with interventions has the potential to promote autonomous motivation [21] and is consistent with the way in which people are accustomed to using the Internet. However, conventional tailoring may still be required to ensure that users are not presented with material or elements that they find so alienating or demotivating that they simply cease using the intervention—for example, the images of older people that younger participants could not relate to in our study, or the German accents that our Austrian participants found off-putting.

### Practice Implication

These findings have clear implications for those who develop health-related websites and digital interventions; these can and should be designed to be accessible and engaging for people with a wide range of levels of health literacy, to help to overcome the “digital divide” and reduce health inequalities. This can be achieved by following the established good design principles we drew on and then using findings from iterative qualitative research to maximize the perceived relevance, credibility, and feasibility of the intervention for different members of the target population. Incorporating interactive and audiovisual elements to increase interest and engagement may also be useful but requires careful development to ensure that they are appropriate for people of both genders, different ages, and from different cultures. Finally, with regard to international dissemination of digital interventions, our findings indicate that the acceptability of interventions is likely to be similar across different countries but can also be improved by making modifications on the basis of feedback from interviews to increase the perceived relevance to the specific cultural context.

## References

[1] Greenhalgh T (2015). Health literacy: Towards system level solutions. BMJ.

[2] Sørensen K, Van den Broucke S, Fullam J, Doyle G, Pelikan J, Slonska Z, Brand H, (HLS-EU) Consortium Health Literacy Project European (2012). Health literacy and public health: A systematic review and integration of definitions and models. BMC Public Health.

[3] Nutbeam D (2008). The evolving concept of health literacy. Soc Sci Med.

[4] Pereira K, Phillips B, Johnson C, Vorderstrasse A (2015). Internet delivered diabetes self-management education: A review. Diabetes Technol Ther.

[5] Thoolen B, de Ridder RD, Bensing J, Gorter K, Rutten G (2007). Who participates in diabetes self-management interventions?: Issues of recruitment and retainment. Diabetes Educ.

[6] Muñoz RF (2010). Using evidence-based internet interventions to reduce health disparities worldwide. J Med Internet Res.

[7] McAuley A (2014). Digital health interventions: Widening access or widening inequalities?. Public Health.

[8] Kusec S, Brborovic O, Schillinger D (2003). Diabetes websites accredited by the Health On the Net Foundation Code of Conduct: Readable or not?. Stud Health Technol Inform.

[9] Sarkar U, Karter A, Liu J, Adler NE, Nguyen R, Lopez A, Schillinger D (2010). The literacy divide: Health literacy and the use of an internet-based patient portal in an integrated health system—Results from the diabetes study of northern California (DISTANCE). J Health Commun.

[10] Glasgow R, Strycker L, Kurz D, Faber A, Bell H, Dickman JM, Halterman E, Estabrooks PA, Osuna D (2010). Recruitment for an internet-based diabetes self-management program: Scientific and ethical implications. Ann Behav Med.

[11] Clement S, Ibrahim S, Crichton N, Wolf M, Rowlands G (2009). Complex interventions to improve the health of people with limited literacy: A systematic review. Patient Educ Couns.

[12] Ryan R, Prictor M, McLaughlin K, Hill S (2008). Audio-visual presentation of information for informed consent for participation in clinical trials. Cochrane Database Syst Rev.

[13] Mackert M, Champlin S, Holton A, Muñoz I, Damásio M (2014). eHealth and health literacy: A research methodology review. J Comput-Mediat Comm.

[14] Jacobs R, Lou J, Ownby R, Caballero J (2014). A systematic review of eHealth interventions to improve health literacy. Health Informatics J.

[15] Barry M, D'Eath M, Sixsmith J (2013). Interventions for improving population health literacy: Insights from a rapid review of the evidence. J Health Commun.

[16] Welch G, Shayne R (2006). Interactive behavioral technologies and diabetes self-management support: Recent research findings from clinical trials. Curr Diab Rep.

[17] Glasgow R (2010). Interactive media for diabetes self-management: Issues in maximizing public health impact. Med Decis Making.

[18] Fransen M, von WC, Essink-Bot M (2012). Diabetes self-management in patients with low health literacy: Ordering findings from literature in a health literacy framework. Patient Educ Couns.

[19] Hirsh D, Clerehan R, Staples M, Osborne R, Buchbinder R (2009). Patient assessment of medication information leaflets and validation of the Evaluative Linguistic Framework (ELF). Patient Educ Couns.

[20] Rotondi A, Eack S, Hanusa B, Spring M, Haas G (2015). Critical design elements of e-health applications for users with severe mental illness: Singular focus, simple architecture, prominent contents, explicit navigation, and inclusive hyperlinks. Schizophr Bull.

[21] Yardley L, Morrison L, Bradbury K, Muller I (2015). The person-based approach to intervention development: Application to digital health-related behavior change interventions. J Med Internet Res.

[22] Williams S, Yardley L, Wills G (2013). A qualitative case study of LifeGuide: users' experiences of software for developing Internet-based behaviour change interventions. Health Informatics J.

[23] Rowsell A, Muller I, Murray E, Little P, Nutbeam D, Yardley L (2015). Patient Views and Experiences of Web-Based Support for Diabetes Self-Management Designed for People with Different Levels of Health Literacy.

[24] van den Haak MJ, de Jong M, Schellens P (2007). Evaluation of an informational web site: Three variants of the think-aloud method compared. Tech Commun.

[25] Chew L, Bradley K, Boyko E (2004). Brief questions to identify patients with inadequate health literacy. Fam Med.

[26] Sørensen K, Van den Broucke S, Pelikan J, Fullam J, Doyle G, Slonska Z, Kondilis B, Stoffels V, Osborne RH, Brand H, HLS-EU Consortium (2013). Measuring health literacy in populations: Illuminating the design and development process of the European Health Literacy Survey Questionnaire (HLS-EU-Q). BMC Public Health.

[27] Braun V, Clarke V (2006). Using thematic analysis in psychology. Qual Res Psychol.

[28] Joffe H, Yardley L, Marks D, Yardley L (2004). Content and thematic analysis. Research Methods for Clinical and Health Psychology.

[29] Charmaz K, Henwood K (2008). Grounded theory. The Sage Handbook of Qualitative Research in Psychology.

[30] Longo D, Schubert S, Wright B, LeMaster J, Williams C, Clore J (2010). Health information seeking, receipt, and use in diabetes self-management. Ann Fam Med.

[31] Smith S, Trevena L, Nutbeam D, Barratt A, McCaffery K (2008). Information needs and preferences of low and high literacy consumers for decisions about colorectal cancer screening: Utilizing a linguistic model. Health Expect.

[32] Yardley L, Morrison LG, Andreou P, Joseph J, Little P (2010). Understanding reactions to an internet-delivered health-care intervention: Accommodating user preferences for information provision. BMC Med Inform Decis Mak.

[33] Ezendam NP, Alpay LL, Rövekamp TA, Toussaint PJ (2005). Experimenting with case-based reasoning to present educative health information on the Internet: The example of SeniorGezond. Stud Health Technol Inform.

[34] Mackert M, Kahlor LA, Tyler D, Gustafson J (2009). Designing e-health interventions for low-health-literate culturally diverse parents: Addressing the obesity epidemic. Telemed J E Health.

[35] Lorig K, Holman H (2003). Self-management education: History, definition, outcomes, and mechanisms. Ann Behav Med.

